# Gender Matters: Workplace Bullying, Gender, and Mental Health

**DOI:** 10.3389/fpsyg.2020.560178

**Published:** 2020-10-06

**Authors:** Michael Rosander, Denise Salin, Lina Viita, Stefan Blomberg

**Affiliations:** ^1^Department of Behavioural Sciences and Learning, Linköping University, Linköping, Sweden; ^2^Department of Management and Organisation, Hanken School of Economics, Helsinki, Finland; ^3^Occupational and Environmental Medicine Center, Department of Health, Medicine and Caring Sciences, Linköping University, Linköping, Sweden

**Keywords:** workplace bullying, gender, mental health, longitudinal study, probability sample, prevalence, measurement

## Abstract

The aim of this study was to examine the role of gender in the process of workplace bullying. In particular, we examined how gender affects reported prevalence rates and health consequences of bullying. In addition, we pay particular attention to if the measurement method – self-labelling or behavioural experience methods – affects potential gender differences. A longitudinal study, with two measurement points 18 months apart, was conducted in Sweden (*n* = 1854 at T1; *n* = 1096 at T2). It was a probability sample out of a population of all 3.3 million people in Sweden working at workplaces with ten or more employees. The results showed a slightly higher tendency for women to self-label as bullied (8% vs. 6%), while a higher proportion of men than women could be labelled as bullied based on the negative acts they had been exposed to (21% vs. 14%). Exposure to negative acts was associated with more subsequent mental health problems for both men and women, whereas self-labelling was associated with mental health problems for men only. Mental health problems at baseline also increased the risk of bullying for both men and women; however, the measurement method affected if the effect was stronger for men or women. Overall, the study advances our understanding of the role of gender in bullying, in particular highlighting the importance of the measurement method for understanding such gender differences.

## Introduction

There is a wide agreement that workplace bullying is a serious work environment problem that has severe consequences for the exposed (Nielsen and Einarsen, 2012, 2018; Arenas et al., 2015; Nauman et al., 2019; Nielsen et al., 2019; Reknes et al., 2019). However, there is less agreement about the causality, the process, and possible moderators and mediators (Nielsen and Einarsen, 2018). In particular, when it comes to gender and gender differences in regard to causality and process results are mixed and inconclusive (Salin et al., 2013; Salin, 2018), calling for further research to understand the significance of gender in the bullying process. The current study contributes with new knowledge on gendered aspects of both exposure and the consequences of workplace bullying. We also investigate and contrast the two most commonly used methods of measurement of workplace bullying (Nielsen et al., 2020b) as the choice of measurement method may affect gender differences found.

As for the prevalence of bullying, many studies report no gender differences at all (e.g., Giorgi et al., 2014; Tsuno et al., 2015), while others find differences (e.g., Cunniff and Mostert, 2012; Giorgi et al., 2013). A recent meta-analysis on the gender of the victims showed that women were over-represented (Zapf et al., 2020). However, the authors noted that the fact that women were also substantially over-represented in the total sample, consisting of 55 studies, largely could explain this difference between men and women. Nevertheless, a majority of the studies showing gender differences indicate that more women are bullied than men. In a review of research on gender and workplace bullying (Salin, 2018) found that a majority of the included studies point to women being the most exposed. However, there are examples of studies showing men as the most exposed to workplace bullying (see Salin, 2018; Rosander and Blomberg, 2019). The results are inconclusive and mixed also when it comes to gender differences in the consequences of bullying. Some studies have found stronger effects for women and some stronger effects for men, while other studies have found no gender differences (for a review see Salin, 2018). The heterogeneity of the different samples suggests a search for moderators. One potential moderator is the way how bullying is measured.

Workplace bullying is defined as systematic negative treatment of an individual over an extended time in situations which he or she has difficulties to defend against (Einarsen et al., 2020). When assessing bullying most studies have used one of two methods, or both in combination: (a) the self-labelling method, involving people assessing if they feel they have been victimised based on their own understanding of the concept of bullying, or based on a given definition; and (b) the behavioural experience method, which entails the perception of being exposed to a range of different bullying behaviours without ever mentioning bullying (Nielsen et al., 2010b). The most commonly used inventory is the Negative Acts Questionnaire (NAQ; Einarsen et al., 2009), but different methods and combinations of methods have been used in research on workplace bullying. This makes it challenging to compare different studies, but not necessarily to compare men and women using the same method and criteria. A pattern that can be discerned is that a majority of studies reporting higher bullying rates for women used self-labelling as method to measure workplace bullying, while studies reporting higher bullying rates for men, although a lot fewer, typically used the behavioural experience method (cf. Salin, 2018). Similarly, with respect to consequences of bullying, it has been suggested that the measurement method may possibly affect if and what kind of gender differences are found (Hoel et al., 2004). Thus, this suggests that the choice of measurement method may be important when studying the role of gender in bullying.

The vast majority of studies on workplace bullying do not have a focus on gender or gender differences, and if gender is part of a study it often has, as Salin (2018) called it, a “gender-as-variable approach.” In many studies gender is controlled for (e.g., Nielsen et al., 2017) adjusting for the possible influence of gender rather than actively examining it. Some studies have focussed on gender differences in terms of consequences (e.g., Glambek et al., 2018), others on differences in risk or protective factors, for example, social support (Nielsen et al., 2020a), or how bullying is construed (Escartín et al., 2011). In this study we investigated how gender in relation to workplace bullying can be understood, both in terms of prevalence and consequences for mental health, and how different measures of bullying, that is, self-labelling versus behavioural experience methods, may affect gender differences found.

The study contributes to the understanding of bullying and gender by examining the role of gender in a national, large-scale probability sample. Also, we examine the role of gender in the association between bullying and mental health using a longitudinal design, in contrast to previous studies that have typically been cross-sectional. A further strength of the study is that it includes two different methods of measuring bullying in the same study, allowing to examine how the measurement method affects gender differences found.

## Gender Differences in Workplace Bullying

In the uncertainty of the diverse results regarding gender differences there are, however, indications that women self-label as bullied to a higher degree than men (Salin, 2003). It has been argued that one possible reason women are more likely to self-label as bullied is related to women having lower social power (Miner and Eischeid, 2012; Salin, 2018). In an unequal situation, the one in power may try to maintain the inequality by more or less openly discriminating and using various negative acts against the less powerful (Sidanius et al., 2004). Studies have also found that women in a higher managerial position are more likely to be bullied (Hoel et al., 2001). Salin (2001, 2003) suggested that there might be a relationship between women and formal position, that is, women are more often bullied than men if in a managerial position. A possible explanation is that because women are in a minority position as managers, they therefore become more salient and vulnerable. Indications of this were also reported by Hoel et al. (2001) showing that almost 16% of female senior managers reported to have been bullied compared to around 6% for male senior managers. Being in a minority position at work is something that has been reported as a possible reason behind higher exposure for men in women dominated occupations, for example, nursing (Eriksen and Einarsen, 2004), public servants (Wang and Hsieh, 2015), and childcare (Lindroth and Leymann, 1993).

Another explanation for a higher level of self-labelling as bullied could be connected to the job itself. Although inconclusive support for the phenomenon of a “glass cliff” (e.g., Adams et al., 2009; Cook and Glass, 2014; Mulcahy and Linehan, 2014; Bechtoldt et al., 2019), it might be a contributing factor to female leaders being vulnerable to bullying. According to the notion of a glass cliff, female leaders are more likely to be chosen for positions associated with poor performance and men more likely to be chosen for positions that are associated with successful performance (Haslam and Ryan, 2008; Bruckmüller et al., 2014; Cook and Glass, 2014). This might entail women to be more vulnerable to criticism due to a hard task, a high risk of failure and poor conditions. This could be further enhanced by stereotypes that women are not suitable for leadership positions (Mulcahy and Linehan, 2014). According to descriptive gender stereotypes women do not have the characteristics required to take on a male-typed job (Heilman, 2001), which could further explain women’s exposure to bulling. If women are successful in male-typed occupations they might face derogation and rejection (Heilman, 2001). Also, women who exhibit stereotypically masculine behaviour and appearance can be at risk. According to Leskinen et al. (2015) women who deviated from traditional gender stereotypes were disproportionately targeted with gender harassment.

The *higher* likelihood of women self-labelling as bullied is of course relative. It could as well be construed as a *lower* likelihood of men to self-label. A lower tendency to self-label for men could be understood, in terms of gender role theory (Eagly and Wood, 2016), as a threat to a man’s masculinity if he was to be viewed as weak and a victim. That and the shame connected to be viewed as vulnerable (Lewis, 2004) could explain this lower likelihood.

Ólafsson and Jóhannsdóttir (2004) reported a rather high agreement between reports of self-labelled bullying and exposure to bullying behaviours (around 85% agreement). That is an overall estimate (including both men and women). They found no overall gender difference in self-labelled bullying, however, men reported being exposed to more bullying behaviours. Gender differences were discussed in terms of how bullying is construed and differences in coping strategies. Men were more likely to confront the bully, while women sought help or support more often when exposed to bullying behaviours. Whereas Ólafsson and Jóhannsdóttir (2004) found a high agreement between self-labelling and exposure to bullying behaviours Salin (2001) found inconsistencies, mainly for lower frequency self-judgements. Neither Salin (2001) nor Ólafsson and Jóhannsdóttir (2004) investigated gender differences comparing different methods of measurement. The current study will extend previous research by examining gender differences in inconsistencies between ways of measuring bullying.

In terms of assessing workplace bullying, Nielsen et al. (2012) concluded that self-labelling as a victim was a better predictor of subsequent increase in mental health problems, compared to just being exposed to bullying behaviours. They did not investigate gender as a possible moderator. Salin (2015) argued for the use of self-labelling, as it also captures the targets’ judgement about whether they can defend themselves against the negative acts or feel victimised by the behaviour, something not captured by the behavioural classification method, otherwise described as a more objective way of measuring workplace bullying (Nielsen et al., 2010b). However, studying gender and bullying only using self-labelling may be problematic in that there are indications showing gender differences in how bullying is interpreted (Escartín et al., 2011). Escartín et al. (2011) found that in their conceptualisations of bullying women were more likely to include person-related forms of bullying, such as emotional abuse, and also rated a number of negative acts as more severe than men did (e.g., social isolation). In a similar vein, Simpson and Cohen (2004) found that women were more likely to perceive certain behaviours as threatening compared to men. Based on this, if there are gender differences in how bullying is construed, using the self-labelling method, men and women would answer slightly different questions, or at least on a different scale. The specific negative acts covered by the behavioural experience method may, of course, also be construed differently. Also, aspects such as ability to defend oneself if exposed is not normally part of the measure when using this method, although Rosander and Blomberg (2019) suggested a measure for that as an addition to the behavioural experience method. In all, each method has shortcomings which would speak for using a combination of both methods when studying bullying and gender.

## Gender Differences in Consequences of Workplace Bullying

The consequences of being bullied have been widely studied. In a meta-analysis based on almost 80 independent samples and about 140,000 participants Nielsen and Einarsen (2012) showed robust evidence for negative consequences of workplace bullying, for example, regarding mental health. Workplace bullying is a strong social stressor, so it is not surprising that there are consequences (Hauge et al., 2010; Nielsen et al., 2012). Whether there are gender differences for these consequences is less certain. As with studies on the prevalence, although not as many, some studies show no gender differences, while others do (Salin, 2018). An example showing gender differences for the outcome is a study by Glambek et al. (2018) who investigated gender differences in the association between exposure to bullying behaviours and neck pain. The level of neck pain was high for women regardless of exposure to bullying behaviours – for men it increased significantly with exposure and for high levels of bullying the baseline differences in pain was nullified. As mentioned above a managerial position can be precarious for women in terms of exposure to bullying behaviours. Such a position can also have an impact on the consequences of uncivil treatment for women. Holmvall and Sobhani (2019) showed that although no gender difference in exposure was found, the consequences in terms of, for example, enthusiasm and feeling at ease was lower for women than men when exposed.

Looking at mental health problems there seem to be some gender differences. Hoel et al. (2004) showed that the risk for mental health consequences differ between men and women depending on the type of bullying behaviour one is exposed to. For men there were a five to six times increased risk when exposed to behaviours such as persistent criticism and being ignored or excluded. For women the corresponding risks were found for negative acts such as hints that one should quit one’s job, being pressured to not claim things one is entitled to, or having allegations made against oneself. In a 5-year prospective study Einarsen and Nielsen (2015) found mental health consequences still lingering long after a target had been exposed to bullying behaviours. However, this was the case only for men who reported a fourfold risk of psychological distress 5 years later. There were no gender differences for the association between self-labelled bullying and mental health in this study.

In terms of criteria for bullying, and of discrepancies in exposure to bullying behaviours and self-labelling, Vie et al. (2011) showed that there are negative consequences of exposure to bullying behaviours regardless if it is labelled as bullying or not. Rosander and Blomberg (2019) showed that already those in risk of being bullied, that is, people that are not exposed on a weekly basis and do not self-label as bullied, but still are the target of a number of negative acts at least now and then, report more health and mental health problems than those who are not bullied.

There are a number of studies that not only have found an association between bullying and subsequent mental health problems, but also findings indicating a reversed relationship, that is, that mental health problems predict subsequent exposure to bullying behaviours (Finne et al., 2011; Nielsen and Einarsen, 2012, 2018; Einarsen and Nielsen, 2015). The reasons for this reversed causality could be that, for example, someone that is depressed views the world more negatively and thereby also evaluates their interactions with others more negatively (Kompier and Taris, 2011). It could also be that a person that has mental health problems cannot always meet the expectations of colleagues or managers and may therefore be at risk of negative treatment (Einarsen, 2000). The reversed relationship has not previously been investigated from a gender perspective, so the current study will add new knowledge to what is known about this relationship.

## Aim and Research Questions

The aim of this study was to longitudinally investigate gender differences in workplace bullying. We studied inconsistences between two ways of reporting exposure (self-labelling vs. behavioural experience), as well as how gender and measurement method affected the relationship between workplace bullying and mental health. The research questions were:

(1) Are there gender differences in the reported prevalence of bullying and are there (gendered) differences and inconsistencies when comparing self-labelled bullying and the behavioural experience method?
(2) Are there gender differences in the association between (a) reported self-labelled bullying, and (b) reported exposure to bullying behaviours at baseline, and mental health problems at follow-up (18 months later)?
(3) Are there gender differences for reversed causality, that is, the association between mental health problems at baseline and (a) reported self-labelled bullying, and (b) reported exposure to bullying behaviours at follow-up (18 months later)?

## Materials and Methods

### Design and Sample

The study had a longitudinal design with two measurement points about 18 months apart. It was a probability sample out of a population of all 3.3 million people in Sweden 18–65 years old working at workplaces with ten or more employees. The sample was drawn by the government agency Statistics Sweden^1^. The baseline data (T1) were collected in the Autumn of 2017 (*n* = 1854) and follow-up data (T2) in the Spring of 2019 (*n* = 1096). Only those responding at T1 were invited to participate at T2. For research question 1 (reported prevalence) all respondents responding at T1 were included. However, for research questions 2 and 3 only the 1096 people that responded both times were included. At baseline, women made up 57% of the sample, and at follow up 58%. The majority had some university or college education (52%; men 42%, women 60%); 44% had 10–12 years of education (men 51%, women 37%), while the rest (4%; men 7%, women 3%) had 9 years or less. About half of the participants (52%) had at least one child (men 54%, women 50%); 54% were married (men 53%, women 55%); 14% had some kind of managerial or supervisory position (men 18%, women 12%). The mean age was 49.3 years (*SD* = 10.0), exactly the same for men and women in the sample. There were some significant differences between men and women in the sample—-women were more highly educated, χ^2^(2) = 39.8, *p* < 0.001, but not as many were in a managerial or supervisory position, χ^2^(1) = 9.6, *p* = 0.002, and the mean income was considerably lower for women. The mean yearly income for women was 384 thousand Swedish krona (tSEK), *SD* = 150, and for men 493 tSEK, *SD* = 254, *t*(1094) = 8.9, *p* < 0.001.

### Measures

Workplace bullying was measured using both a self-labelling method based on a definition and a behavioural experience method. The self-labelling method consisted of one item asking respondents if they had been bullied at work during the past 6 months. The question came right after a definition of bullying saying that “Bullying occurs when a person, systematically over time, is subjected to negative treatment by one or more persons, in situations where the victim has difficulties defending him- or herself.” Added to that it was pointed out that “It is not bullying if two equally strong people are in conflict with each other.” Answers were given on a five-point scale from Never, Now and then, Monthly, Weekly, and Daily. The question is part of the PSYWEQ questionnaire (Rosander and Blomberg, 2018, 2019; Blomberg and Rosander, 2020). A few pages before the self-label question we incorporated the Negative Acts Questionnaire–Revised (NAQ–R; Einarsen et al., 2009) in the PSYWEQ. The NAQ–R consists of 22 negative behaviours one can be exposed to at work covering both work- and person-related acts, and uses the same frequency scale as the single-item self-labelling question described above. Cronbach’s alpha for the NAQ–R was.89 at T1, and.90 at T2. In this study, when categorising bullying, we used the cut-off score for the NAQ–R suggested by Notelaers and Einarsen (2013) in which the lower cut-off, “Occasional bullying,” is the NAQ–R sum score equal to or larger than 33. Self-labelling as bullied at least now and then was used as the cut-off for the self-labelling method.

Mental health was measured using the Hospital Anxiety and Depression Scale (HADS; Zigmond and Snaith, 1983). The HADS consists of 14 items, half of which measures anxiety and half depression symptoms, on a scale from 0 to 3. A sample item is “I feel cheerful” with possible responses from “Not at all” to “Most of the time”. Cronbach’s alpha for the HADS was 0.90 at T1 and 0.89 at T2.

Demographic variables included gender, education, income, and managerial position. Gender (man or woman) was taken directly from the Swedish population register, meaning respondents did not have the possibility to self-categorise as ‘other’ or differently from their legal gender. Education was a classification taken from the population register of Sweden consisting of eight levels of education, less than 9 years, 9–10 years, 11–12 years, 1 year at the university, 2 years at the university, 3 years at the university, four or more years at the university, and as the eighth and final category, doctoral education. Similarly, income (operationalised as yearly income) was taken from the population register of Sweden. The variable Managerial position was a question in the PSYWEQ (Rosander and Blomberg, 2018) asking respondents if they had some kind of managerial or supervisory position at work (Yes/No). The reason for including position was that women often are in a minority position when in a supervisor or managerial position and thereby possibly at higher risk of being bullied (Salin, 2001). As shown above there were significant differences between men and women in the sample regarding education, income and position.

### Statistical Analyses

All analyses were conducted using IBM SPSS version 26. In order to test inconsistencies in responses across self-labelled bullying and behavioural experiences methods we used McNemar tests. When only focussing on the inconsistent cases we tested gender differences using a χ^2^-test. When investigating gender as a moderator Hayes’ PROCESS macro for SPSS (version 3.4) was used (Hayes, 2018). We adjusted for the baseline of the dependent variable in all moderation analysis to more clearly show the causal order. The differences between men and women for the demographic variables (education, income and managerial position) and differences in mental health in a study on gender need to be handled with care. If merely controlling for variables that also constitute important differences in a gendered perspective, we would remove variance connected to these aspects and at the same time risk inflating biological differences (Spector and Brannick, 2011; Becker et al., 2016). As recommended by Becker et al. (2016) we did the analyses first without the control variables except for the baseline of the dependant variable. We then compared the results to analyses where we adjusted for mental health and controlling for education, income, and managerial position. If there were no differences regarding the independent variable and interaction when adding the control variables only the results from the first analysis was reported (Becker, 2005).

### Ethical Considerations

The project was approved by the Regional Ethical Review Board at Linköping University, Sweden. Protocol number: 2017/336-32. All participant information was handled only by Statistics Sweden. Statistics Sweden sent out the questionnaires, however, they did not handle any of the responses. The research group handled the responses but had only access to a code for each individual to ensure anonymity of the participants. The invitation to participate included information on research ethics, that participation was voluntary, that all aspects of the study was handled with confidentiality, and that participants had the right to withdraw even if already having sent in the answers (together with information on how to do that).

## Results

In Table 1 the means, standard deviations, and intercorrelations for all study variables are presented (for the dichotomous variables and self-labelled bullying, proportions are presented). Of the respondents at T1 (*n* = 1849), 7% self-labelled as bullied at least now and then (8% women and 6% men, the difference was not significant). Using the behavioural experience method, 17% scored over the lower cut-off score at 33 of the NAQ–R suggested by Notelaers and Einarsen (2013) (14% women and 21% men). This difference between women and men was significant, χ^2^(1) = 15.7, *p* < 0.001.

**TABLE 1:** Descriptive statistics and intercorrelations for the variables of the study (*n* = 1096).

	**Mean**	***SD***	**1.**	**2.**	**3.**	**4.**	**5.**	**6.**	**7.**	**8.**	**9.**
(1) Gender	58% women										
(2) Education	4.72	1.72	0.15***								
(3) Income^†^	429	207	−0.26***	0.33***							
(4) Position	14% managers		−0.09**	0.12***	0.45***						
(5) HADS T1	0.63	0.47	0.08**	0.02	−0.09**	−0.07*					
(6) HADS T2	0.61	0.45	0.07*	–0.00	−0.08**	−0.08*	0.68***				
(7) NAQ T1	1.25	0.33	−0.06*	–0.00	−0.06*	–0.02	0.48***	0.35***			
(8) NAQ T2	1.20	0.30	–0.05	0.02	–0.01	–0.00	0.38***	0.44***	0.60***		
(9) Bullying T1	5% bullied		0.06*	–0.04	−0.09**	−0.07*	0.19***	0.11***	0.29***	0.16***	
(10) Bullying T2	5% bullied		–0.02	–0.01	–0.00	0.00	0.15***	0.20***	0.24***	0.33***	0.29***

Bullying is self-labelled bullying, proportion is self-labelling as bullied at least now and then. ^†^Yearly income in thousands of Swedish krona (SEK).*p < 0.05 **p < 0.01 ***p < 0.001.

Of the women who self-labelled as bullied (at least now and then) at T1 37% were not bullied according to the lower cut-off score of the NAQ–R. Their mean NAQ–R score was 27.9 (*SD* = 3.3). For men who self-labelled as bullied the corresponding percentage was 22% (NAQ–R mean = 28.2, *SD* = 2.1). Of those scoring over the lower cut-off score for the NAQ-R, 10% of the women did not self-label as bullied (NAQ–R mean = 37.6, *SD* = 5.7). The corresponding percentage for men was 17% (NAQ–R mean = 38.6, *SD* = 7.4). McNemar tests for inconsistency regarding self-labelling and the behavioural experience method of measuring bullying showed inconsistencies for both women, χ^2^(1) = 31.2, *p* < 0.001, and men, χ^2^(1) = 95.8, *p* < 0.001. However, only focussing on those who reported inconsistencies showed a significantly larger percentage of women (24%) than men (9%) self-labelling as bullied, but not being over the lower cut-off of the NAQ–R, χ^2^(1) = 10.9, *p* = 0.001. For the reverse – over the cut-off of the NAQ–R, but not self-labelling as bullied – there were no statistically significant gender differences although a somewhat larger percentage of men than women showed this inconsistency.

### Gender and the Association Between the Two Methods of Measurement

Possible gendered differences between the self-labelling and the behavioural experience methods were tested using moderation analyses. First, the association between self-labelled bullying at baseline and exposure to bullying behaviours at follow-up, then the reverse association. In both analyses gender was added as a moderator investigating the interaction with the predictor.

**FIGURE 1:**
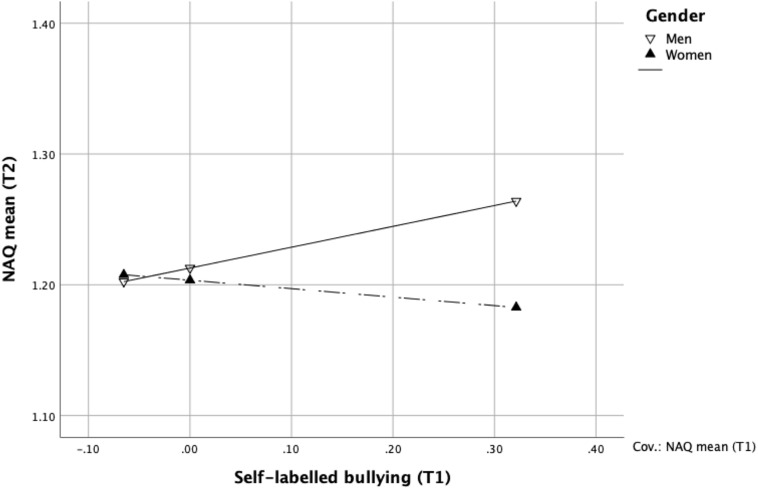
The interaction between self-labelled bullying at T1 and gender with regard to exposure to bullying behaviours at T2, *n* = 1089.

In a moderation analysis controlling for baseline exposure to bullying behaviours (Table 2), gender significantly moderated the association between self-labelled bullying at baseline and exposure to bullying behaviours at follow-up (*b* = –0.22; 95% CI –0.33, –0.12). In Figure 1 this interaction is presented showing a significant positive interaction for men (*b* = 0.16; 95% CI 0.06, 0.25) while the association for women was negative (*b* = –0.06; 95% CI –0.12, –0.01). This analysis was repeated also controlling for education, income, position, and mental health at baseline—-as all four variables showed significant differences between men and women, but the results were essentially identical. This is why only the results of the first analysis is presented in Table 2 as suggested by Becker (2005).

**TABLE 2:** Moderation analysis predicting exposure to bullying behaviours at T2.

	***b***	***SE* b**	**95% CI b**	
Self-labelled bullying (T1)	0.16	0.05	[0.06; 0.25]	*p* < 0.001
Gender	–0.01	0.01	[–0.04; 0.02]	*p* = 0.529
Self-labelled bullying (T1) × Gender	–0.22	0.05	[–0.33; –0.12]	*p* < 0.001
NAQ–R (T1)	0.54	0.02	[0.50; 0.59]	*p* < 0.001

*Dependent variable: NAQ–R (T2).*

We also investigated whether the reversed condition, that is, if gender moderated the association between exposure to bullying behaviours at baseline and self-labelled bullying at follow-up adjusting for self-labelled bullying at baseline. The results showed a significant direct effect of exposure to bullying behaviours at baseline on self-labelled bullying at follow-up (*b* = 0.20; 95% CI 0.12, 0.28), but no significant interaction (Table 3). We repeated the analysis also controlling for education, income, position, and mental health at baseline. The result was essentially identical.

**TABLE 3:** Moderation analysis predicting self-labelled bullying at T2.

	***b***	**SE b**	**95% CI b**	
NAQ–R (T1)	0.21	0.04	[0.13; 0.29]	*p* < 0.001
Gender	0.00	0.02	[–0.03; 0.04]	*p* = 0.865
NAQ–R (T1) × Gender	0.03	0.06	[–0.08; –0.14]	*p* = 0.636
Self-labelled bullying (T1)	0.21	0.03	[0.14; 0.27]	*p* < 0.001

*Dependent variable: Self-labelled bullying (T2).*

### Gender, Bullying, and Mental Health Problems

**FIGURE 2:**
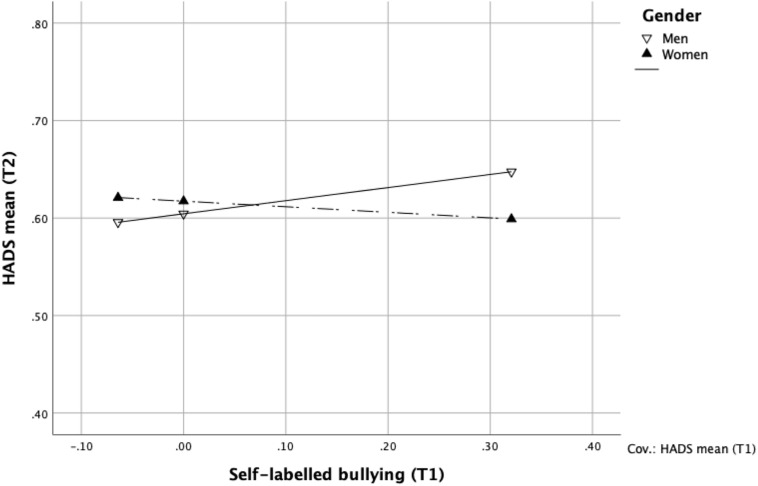
The interaction between self-labelled bullying at T1 and gender with regard to mental health problems at T2, *n* = 1076.

Moderation analyses were conducted to determine the interaction between bullying at baseline and gender with regard to mental health at follow-up. First, the interaction between self-labelled bullying and gender (Table 4), and then the same analysis, but with exposure to bullying behaviour instead of self-labelled bullying as independent variable. Both analyses were repeated adding education, income, and position as controls for comparison with the original analysis. An analysis adjusting for baseline mental health showed that gender moderated the association between level of self-labelled bullying at T1 and mental health at T2 (*b* = –0.19; 95% CI –0.34, –0.05). As displayed in Figure 2, men reported a decrease in mental health at follow-up (*b* = 0.13; 95% CI 0.01, 0.26) whereas there for women was no association between self-labelled bullying at baseline and mental health at follow-up (*b* = –0.06; 95% CI –0.13, 0.01). The result of the repeated analysis showed an essentially identical result. The second analysis, in which the independent variable was exposure to bullying behaviours at T1 instead of self-labelled bullying, showed no significant interaction between exposure to bullying behaviours at baseline and gender with regard to mental health at follow-up adjusting for mental health at baseline (Table 5). There was no gender difference, and no direct effect of baseline exposure to bullying behaviours on mental health at follow-up. The zero-order correlation between baseline exposure to bullying behaviours and mental health at follow-up 18 months later was significant (*r* = 0.35, *p* < 0.001).

**TABLE 4:** Moderation analysis predicting mental health problems at T2.

	***b***	***SE* b**	**95% CI b**	
Self-labelled bullying (T1)	0.13	0.07	[0.01; 0.26]	*p* = 0.039
Gender	0.01	0.02	[–0.03; 0.05]	*p* = 0.529
Self-labelled bullying (T1) × Gender	–0.19	0.07	[–0.34; –0.05]	*p* = 0.010
HADS (T1)	0.66	0.02	[0.62; 0.70]	*p* < 0.001

*Dependent variable: HADS (T2).*

**TABLE 5:** Moderation analysis predicting mental health problems at T2.

	***b***	***SE* b**	**95% CI b**	
NAQ–R (T1)	0.07	0.05	[–0.03; 0.16]	*p* = 0.156
Gender	0.02	0.02	[–0.02; 0.06]	*p* = 0.353
NAQ–R (T1) × Gender	–0.03	0.06	[–0.15; 0.09]	*p* = 0.613
HADS (T1)	0.64	0.02	[0.59; 0.69]	*p* < 0.001

*Dependent variable: HADS (T2).*

Regardless of exposure to workplace bullying, women reported a higher level of mental health problems, both at baseline, *t*(1089) = –2.63, *p* = 0.009; the mean for men was 0.59 (*SD* = 0.46) and for women 0.67 (*SD* = 0.48), and at follow-up, *t*(1081) = –2.20, *p* = 0.028; the mean for men was 0.57 (*SD* = 0.44) and for women 0.64 (*SD* = 0.46).

**FIGURE 3:**
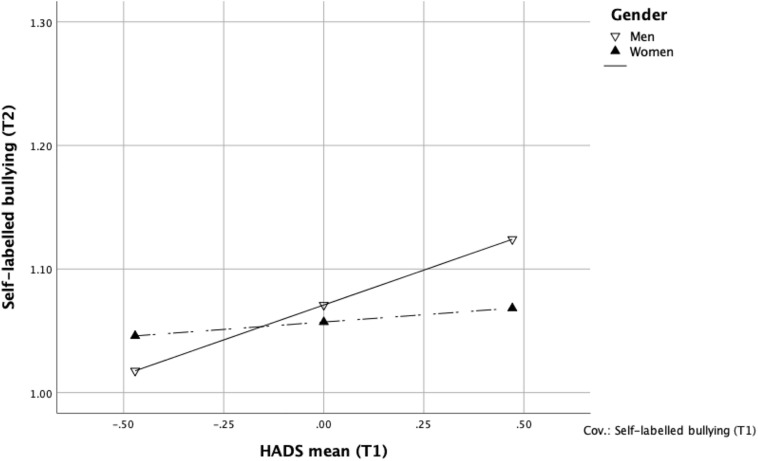
The interaction between mental health problems at T1 and gender with regard to self-labelled bullying at T2, *n* = 1075.

Next, we turn to investigate the reversed causality, that is, the association between mental health problems at baseline and bullying at follow-up. First, predicting self-labelling at T2 adjusting for self-labelling at baseline (see Table 6) showed that gender moderated the association (*b* = –0.09; 95% CI –0.17, –0.01). In Figure 3 the association for men and women is presented. For men, there was a significant association (*b* = 0.11; 95% CI 0.05, 0.17), whereas for women the association was not significant (*b* = 0.02; 95% CI –0.03, 0.07). Repeating the analysis adding education, income, and position as controls showed an essentially identical result. Second, when predicting exposure to bullying behaviours at follow-up adjusting for the exposure at baseline gender was not a moderator (Table 7). There was a significant direct effect of mental health problems at baseline on the exposure to bullying behaviours at follow-up (*b* = 0.10; 95% CI 0.05, 0.15). Also the zero-order correlation for this association was significant (*r* = 0.38, *p* < 0.001).

**TABLE 6:** Moderation analysis predicting self-labelled bullying at T2.

	***b***	***SE* b**	**95% CI b**	
HADS (T1)	0.11	0.03	[0.05; 0.17]	*p* = 0.002
Gender	–0.01	0.02	[–0.05; 0.02]	*p* = 0.459
HADS (T1) × Gender	–0.09	0.04	[–0.17; –0.01]	*p* = 0.024
Self-labelled bullying (T1)	0.30	0.03	[0.25; 0.36]	*p* < 0.001

*Dependent variable: Self-labelled bullying (T2).*

**TABLE 7:** Moderation analysis predicting exposure to bullying behaviours at T2.

	***b***	***SE* b**	**95% CI b**	
HADS (T1)	0.10	0.03	[0.05; 0.15]	*p* < 0.001
Gender	–0.02	0.01	[–0.04; 0.01]	*p* = 0.268
HADS (T1) × Gender	–0.04	0.03	[–0.10; 0.02]	*p* = 0.181
NAQ–R (T1)	0.49	0.03	[0.44; 0.54]	*p* < 0.001

*Dependent variable: NAQ–R (T2).*

## Discussion

This study examined workplace bullying and mental health longitudinally from a gender perspective. The use of two different measurements of bullying provided data on inconsistencies comparing the two methods as well as gender differences with regard to mental health at follow-up. First of all, the findings showed gender *differences in prevalence* depending on the way bullying was measured. For self-labelling there were no significant gender differences (although a slightly higher percentage of women were bullied). However, using the behavioural experience method men reported being significantly more exposed to bullying behaviours than women. There were also *gendered inconsistencies* comparing results from the self-labelling and behavioural experience methods. Almost a quarter of the women who self-labelled as bullied did not report being exposed to bullying behaviours enough to be categorised as bullied using the lower cut-off score of the NAQ–R. For men less than one in ten showed this inconsistency. The reverse inconsistency, reporting being exposed to bullying behaviours over the cut-off score of the NAQ–R while not self-labelling as bullied showed no gender differences. Finding inconsistencies is not unique to the current study, for example, Nielsen et al. (2009) showed that in their sample about 7% answered inconsistently – about 4% were exposed to bullying behaviours, but did not feel bullied, and almost 3% felt bullied, but were not exposed according to the criterion used in their study of at least two weekly negative acts. However, they did not study gender differences.

At first glance maybe an explanation in terms of women being more sensitive and therefore experience being bullied to a higher degree, although being exposed to bullying behaviour to a lesser degree than men, would be tempting. Previous research has shown that in line with social power theory, both women and ethnic minority members, who tend to have both lower power and occupy lower positions in the organisational hierarchy, may feel more intimidated and stressed by negative acts at work (Cortina et al., 2002; Salin, 2018). Lower power may thus sensitise a person to perceived threats (Anderson and Berdahl, 2002; Keltner et al., 2003). Another explanation would be looking at the propensity of men to acknowledge being a victim. Based on gender role theory (Eagly and Wood, 2016) this perceived weakness could be construed as a threat to one’s masculinity. As Lewis (2004) pointed out there is shame connected to showing signs of vulnerability. The stronger association for men between self-labelling at baseline and exposure to bullying behaviours in our results could be understood as different thresholds for men and women for when acknowledging to oneself that a negative treatment actually is bullying. It takes more for a man to acknowledge weakness, and when he does it corresponds to a higher level of exposure to negative behaviours. A different threshold for men and women could also be viewed as a difference in interpretation. Escartín et al. (2011) showed gender differences in how bullying is conceptualised, men and women including different aspects of bullying and also rating the severity differently. Also, Simpson and Cohen (2004) showed gender differences in what is experienced as threatening. The stronger association between self-labelled bullying and subsequent reported exposure to bullying behaviours in our results is consistent with this difference in conceptualisation. Differences in self-labelled bullying could also be found in men’s responses to exposure to negative behaviours. According to social power theory men have a higher social power, which could imply that men in general have a better chance to stop unwanted negative behaviours directed at them, and thereby do not see themselves as victims of bullying to the same degree. Ólafsson and Jóhannsdóttir (2004) found that men more often confronted the bully, however, this does not say men are more successful in stopping the behaviours. This calls for more research—if there are gender differences not only in the actions taken against bullies, but also what the consequences of these actions are.

Another explanation for the discrepancy between self-labelled bullying and exposure to bullying behaviours could be that women include more types of negative acts when self-labelling, such as, for example, sexual harassment and discrimination, and may be more exposed to those types of negative treatments (Nielsen et al., 2010a). As those are not covered in the NAQ–R it is not reflected in the score of exposure to bullying behaviours. However, that cannot explain that there was no association between self-labelled bullying and mental health problems at follow-up for women. If self-labelling would include more negative acts than those captured by NAQ–R the consequences would be as severe or worse also for women.

It is interesting to note that in terms of how the two methods of measuring bullying compare over time, self-labelling as bullied at baseline had a significant positive association to exposure to bullying behaviours at follow-up for men, whereas women actually had a significant negative association. A negative association means a decreasing risk for bullying 18 months later as measured by the behavioural experience method. This could be understood in terms of a higher help-seeking tendency among women when exposed to workplace bullying (Ólafsson and Jóhannsdóttir, 2004). Once a woman self-labels as bullied she may thus be more likely to seek help, resulting in an actual reduction in bullying behaviour. Ólafsson and Jóhannsdóttir also noted differences in coping strategies where men were more likely to confront the bully, while women more often applied for transfer or reported in sick.

Many studies have shown negative consequences of workplace bullying for both men and women in terms of, for example, mental health problems (Nielsen and Einarsen, 2012). In this study we have examined gender differences in this regard – differences in exposure to workplace bullying and the consequences in terms of subsequent mental health problems. The results showed a positive association between self-labelled bullying at baseline and mental health problems at follow-up 18 months later, but only for men. For women there was no significant association, but a higher overall level of mental health problems compared to men. Continuing the reasoning from above, this difference could be understood in terms of a higher threshold for self-labelled bullying for men, that is, it takes more exposure for a man to admit to being bullied. For the same score of self-labelled bullying, the level of exposure to bullying behaviours is higher for men, and the consequences are more serious. Our results are similar to a study of neck pain by Glambek et al. (2018) who also only found an association for men, and a higher level for women regardless of exposure to bullying. Why the consequences for women in terms of mental health problems in our study did not show any association with baseline self-labelled bullying is not clear. In light of the vast number of studies showing an overall positive association between bullying and mental health problems, a positive, but maybe smaller positive association would be expected also for women in this study. Smaller, due to the fact that women reported higher levels of mental health problems overall.

There were gender differences in the association between bullying and subsequent mental health problems, but only when using the self-labelling method. For the behavioural experience method there were no significant gender differences for this association (overall *r* = 0.35). This could mean the differences found for self-labelling are due to a difference in scale, that is, a difference in how the concept of bullying is interpreted by men and by women (Escartín et al., 2011). However, as Hoel et al. (2004) showed, exposure to different bullying behaviours may have different consequences for men and women. In our study there were no big variations in the kind of bullying behaviours men and women reported. Hoel et al. (2004) found, for example, exclusion to result in more severe consequences for men, and getting allegations would be more damaging for women, none of which showed gender differences in our data.

Looking at the reversed causality, that is, the association between mental health problems at baseline and bullying at follow-up, the results also showed gender differences. As before, there were no gender differences in this association for the behavioural experience method, but there was a significant direct effect (*b* = 0.10, *p* < 0.001). However, using the self-labelling method there were gender differences. The results showed that the association between mental health problems at baseline and subsequent self-labelled bullying only was significant for men. The suggested reasons for the reversed relationship involve a more negative evaluation of things that happen to oneself (Kompier and Taris, 2011). Such an evaluation could include interpreting negative behaviours as bullying more easily. It has also been suggested that mental health problems could result in difficulties to live up to expectations at work, both social and task related (Nielsen and Einarsen, 2018). The former explanation does not really imply any gender differences; however, the latter may be a way to understand our results. In terms of gender role theory (Eagly and Wood, 2016) expectations on appropriate behaviours at work differs for men and women, and being weak and vulnerable having mental health problems may to a larger degree violate the expectations of what it means to be a man and could possibly trigger others’ aggressive or excluding behaviours.

### Practical Implications

This study illustrates that the method used to measure bullying affects the results obtained and in particular gender differences found. This points to the importance of combining both measurement methods (cf. Nielsen et al., 2020b). Whereas the behavioural checklist method fails to identify whether targets feel victimised by the acts reported, self-labelling may lead to underestimation because of unwillingness to label as a victim. This points to the importance of including both methods to get a fuller picture.

In particular, there seems to be a higher threshold for men to admit being the victim of bullying. It has possible consequences in the present, in the bullying situation, but more importantly consequences for later well-being. The results showed an association between self-labelling as bullied and later mental health problems, but only for men. The higher threshold for men could also mean that they seek help later on in the process, if at all. When finally coming to terms with being a victim of bullying it could be too late in many cases as workplace bullying is understood as an escalating process. The severity of the consequences is high already at lower levels of exposure and increase as the bullying process escalate (Rosander and Blomberg, 2019).

### Strengths, Limitations and Suggestions for Further Research

A strength of this study is that it is based on a probability sample of the Swedish workforce drawn from the total population of about 3.3 million people working at workplaces of ten or more people. The use of a two-step strategy when it comes to the moderation analyses was also a strength. First, running the analysis only adjusting for the baseline of the dependent variable to clarify the causal order, then repeating the analysis with education, income, position, and mental health at baseline as controls. This allowed us to evaluate the results without risking inflating the biological differences, but at the same time show that the result remained essentially the same also when controlling for a number of demographic variables differentiating men and women. While variables such as gender and income were taken directly from the population register of Sweden, most measures of the study were self-report measures which may have influenced the results. However, alternative methods such as, for example, observational methods or peer nomination methods, would impose problems (Cowie et al., 2002) – both regarding accuracy as it involves highly subjective phenomena, as well as, possible ethical issues. It is also likely that others only hold limited information about another person’s exposure to workplace bullying and mental health, or may be part of the problem being bullies themselves (Salin and Notelaers, 2018). In terms of self-labelled bullying, the self-report is also something that is used as a way to understand the result as previous studies have shown gender differences in how workplace bullying is construed.

This study provides novel insights into the role of gender in workplace bullying. However, it also raises new questions. For instance, bullying has been described as an escalating process (Einarsen, 2000). However, it remains open if the escalation process looks the same for men and women. If there are gender differences, are they similar across different levels of bullying? That is beyond the scope of this study but would be a welcome addition to and continuation of the current study.

## Conclusion

We have investigated gender differences in workplace bullying longitudinally. As previous studies on gender differences in bullying have shown mixed and inconclusive results the current study contributes to the understanding of the significance of gender in the bullying process. The results indicate that gender differences are most prominent in terms of self-labelling as bullied, with men less likely than women to self-label and men reporting stronger relationships between self-labelled bullying and mental health. Overall, the findings highlight the importance of the measurement method, thus providing a potential explanation for some of the inconclusive and mixed results in previous research.

## Data Availability Statement

The raw data supporting the conclusions of this article will be made available by the authors, without undue reservation.

## Ethics Statement

The studies involving human participants were reviewed and approved by the Regional Ethical Review Board at Linköping University. Written informed consent for participation was not required for this study in accordance with the national legislation and the institutional requirements.

## Author Contributions

MR and SB collected the data. MR, LV, and SB were involved in the initial planning of the study. MR analysed the data and initiated and wrote the manuscript. DS contributed to the writing of the manuscript. MR, DS, LV, and SB read, commented on, and approved the final manuscript. All the authors contributed to the article and approved the submitted version.

## Conflict of Interest

The authors declare that the research was conducted in the absence of any commercial or financial relationships that could be construed as a potential conflict of interest. The reviewer DZ declared a past co-authorship with one of the authors DS to the handling editor.

## Footnotes

1 https://www.scb.se/en

## References

[B1] Adams S. M. Gupta A. Leeth J. D. (2009). Are female executives over-represented in precarious leadership positions? *Br. J. Manag.* 20 1–12. 10.1111/j.1467-8551.2007.00549.x

[B2] Anderson C. Berdahl J. L. (2002). The experience of power: examining the effects of power on approach and inhibition tendencies. *J. Pers. Soc. Psychol.* 83 1362–1377. 10.1037/0022-3514.83.6.1362 12500818

[B3] Arenas A. Giorgi G. Montani F. Mancuso S. Perez J. F. Mucci N. (2015). Workplace bullying in a sample of italian and spanish employees and its relationship with job satisfaction, and psychological well-being. *Front. Psychol.* 6:1912. 10.3389/fpsyg.2015.01912 26696948 PMC4678195

[B4] Bechtoldt M. N. Bannier C. E. Rock B. (2019). The glass cliff myth? - Evidence from Germany and the U.K. *Leadersh. Q.* 30 273–297. 10.1016/j.leaqua.2018.11.004

[B5] Becker T. E. (2005). Potential problems in the statistical control of variables in organizational research: a qualitative analysis with recommendations. *Organ. Res. Methods* 8 274–289. 10.1177/1094428105278021

[B6] Becker T. E. Atinc G. Breaugh J. A. Carlson K. D. Edwards J. R. Spector P. E. (2016). Statistical control in correlational studies: 10 essential recommendations for organizational researchers. *J. Organ. Behav.* 37 157–167. 10.1002/job.2053

[B7] Blomberg S. Rosander M. (2020). Exposure to bullying behaviours and support from co-workers and supervisors: a three-way interaction and the effect on health and well-being. *Intern. Archiv. Occup. Environ. Health* 93 479–490. 10.1007/s00420-019-01503-1507 PMC7118028 31828422

[B8] Bruckmüller S. Ryan M. K. Rink F. Haslam S. A. (2014). Beyond the glass ceiling: the glass cliff and its lessons for organizational policy. *Soc. Issues Pol. Rev.* 8 202–232. 10.1111/sipr.12006

[B9] Cook A. Glass C. (2014). Above the glass ceiling: when are women and racial/ethnic minorities promoted to CEO? *Strateg. Manag. J.* 35 1080–1089. 10.1002/smj.2161

[B10] Cortina L. M. Magley V. J. Lim S. G. (2002). “Individual differences in response to incivility in the workplace,” in *Proceedings of the Annual meeting of the Academy of Management*, Denver, CD.

[B11] Cowie H. Naylor P. Rivers I. Smith P. K. Pereira B. (2002). Measuring workplace bullying. *Aggress. Viol. Behav.* 7 33–51. 10.1016/s1359-1789(00)00034-33

[B12] Cunniff L. Mostert K. (2012). Prevalence of workplace bullying of South African employees. *SA J. Hum. Resour. Manag.* 10:450 10.4102/sajhrm.v10i1.450

[B13] Eagly A. H. Wood W. (2016). “Social role theory of sex differences,” in *The Wiley Blackwell Encyclopedia of Gender and Sexuality Studies*, eds Naples N. Hoogland R. C. Wickramasinghe M. Wong W. C. A. (Evanston, IL: Northwestern University), 1–3. 10.1002/9781118663219.wbegss183

[B14] Einarsen S. (2000). Harassment and bullying at work. *Aggress. Viol. Behav.* 5 379–401. 10.1016/s1359-1789(98)00043-43

[B15] Einarsen S. Hoel H. Notelaers G. (2009). Measuring exposure to bullying and harassment at work: validity, factor structure and psychometric properties of the Negative Acts Questionnaire-Revised. *Work Stress* 23 24–44. 10.1080/02678370902815673

[B16] Einarsen S. Hoel H. Zapf D. Cooper C. L. (2020). “The concept of bullying and harassment at work: the European tradition,” in *Bullying and Harassment in the Workplace: Theory, Research and Practice*, 3rd Edn, eds Einarsen S. V. Hoel H. Zapf D. Cooper C. L. (Boca Raton: CRC Press), 3–53. 10.1201/ebk1439804896-3

[B17] Einarsen S. Nielsen M. B. (2015). Workplace bullying as an antecedent of mental health problems: a five-year prospective and representative study. *Intern. Arch. Occup. Environ. Health* 88 131–142. 10.1007/s00420-014-0944-947 24840725

[B18] Eriksen W. Einarsen S. (2004). Gender minority as a risk factor of exposure to bullying at work: the case of male assistant nurses. *Eur. J. Work Organ. Psychol.* 13 473–492. 10.1080/13594320444000173

[B19] Escartín J. Salin D. Rodríguez-Carballeira Á (2011). Conceptualizations of workplace bullying. *J. Pers. Psychol.* 10 157–165. 10.1027/1866-5888/a000048

[B20] Finne L. B. Knardahl S. Lau B. (2011). Workplace bullying and mental distress - a prospective study of Norwegian employees. *Scand. J. Work Environ. Health* 37 276–287. 10.5271/sjweh.3156 21373722

[B21] Giorgi G. Ando M. Arenas A. Shoss M. K. Leon-Perez J. M. (2013). Exploring personal and organizational determinants of workplace bullying and its prevalence in a Japanese sample. *Psychol. Viol.* 3 185–197. 10.1037/a0028049

[B22] Giorgi G. Leon-Perez J. M. Arenas A. (2014). Are bullying behaviors tolerated in some cultures? evidence for a curvilinear relationship between workplace bullying and job satisfaction among Italian workers. *J. Bus. Ethics* 131 227–237. 10.1007/s10551-014-2266-2269

[B23] Glambek M. Nielsen M. B. Gjerstad J. Einarsen S. (2018). Gender differences in the relationship between workplace bullying and subjective back and neck pain: a two-wave study in a Norwegian probability sample. *J. Psychosom. Res.* 106 73–75. 10.1016/j.jpsychores.2018.01.010 29455903

[B24] Haslam S. A. Ryan M. K. (2008). The road to the glass cliff: differences in the perceived suitability of men and women for leadership positions in succeeding and failing organizations. *Leadersh. Q.* 19 530–546. 10.1016/j.leaqua.2008.07.011

[B25] Hauge L. J. Skogstad A. Einarsen S. (2010). The relative impact of workplace bullying as a social stressor at work. *Scand. J. Psychol.* 51 426–433. 10.1111/j.1467-9450.2010.00813.x 20338011

[B26] Hayes A. F. (2018). *Introduction to Mediation, Moderation, and Conditional Process Analysis: A Regression-Based Approach.* New York, NY: Guilford Press.

[B27] Heilman M. E. (2001). Description and prescription: how gender stereotypes prevent women’s ascent up the organizational ladder. *J. Soc. Issues* 57 657–674. 10.1111/0022-4537.00234

[B28] Hoel H. Cooper C. L. Faragher B. (2001). The experience of bullying in Great Britain: the impact of organizational status. *Eur. J. Work Organ. Psychol.* 10 443–465. 10.1080/13594320143000780

[B29] Hoel H. Faragher B. Cooper C. L. (2004). Bullying is detrimental to health, but all bullying behaviours are not necessarily equally damaging. *Br. J. Guidance Counsel.* 32 367–387. 10.1080/03069880410001723594

[B30] Holmvall C. M. Sobhani S. M. (2019). Incivility toward managers: gender differences in well-being outcomes. *Equal. Divers. Inclusion Intern. J.* 39 301–317. 10.1108/edi-07-2018-2120

[B31] Keltner D. Gruenfeld D. H. Anderson C. (2003). Power, approach, and inhibition. *Psychol. Rev.* 110 265–284. 10.1037/0033-295x.110.2.265 12747524

[B32] Kompier M. A. Taris T. W. (2011). Understanding the causal relations between psychosocial factors at work and health - a circular process. *Scand. J. Work Environ. Health* 37 259–261. 10.5271/sjweh.3172 21643622

[B33] Leskinen E. A. Rabelo V. C. Cortina L. M. (2015). Gender stereotyping and harassment: a “catch-22” for women in the workplace. *Psychol. Public Pol. Law* 21 192–204. 10.1037/law0000040

[B34] Lewis D. (2004). Bullying at work: the impact of shame among university and college lecturers. *Br. J. Guidan. Counsel.* 32 281–299. 10.1080/03069880410001723521

[B35] Lindroth S. Leymann H. (1993). *Vuxenmobbning Mot En Minoritetsgrupp Män Inom Barnomsorgen: om mäns Jämställdhet i ett Kvinnodominerat yrke (Bullying Against a Minority Group of Men in Childcare: on Men’s Equality in a Women-Dominated Profession).* Stockholm: Swedish National Board of Occupational Safety and Health.

[B36] Miner K. N. Eischeid A. (2012). Observing incivility toward coworkers and negative emotions: do gender of the target and observer matter? *Sex Roles* 66 492–505. 10.1007/s11199-011-0108-100

[B37] Mulcahy M. Linehan C. (2014). Females and precarious board positions: further evidence of the glass cliff. *Br. J. Manag.* 25 425–438. 10.1111/1467-8551.12046

[B38] Nauman S. Malik S. Z. Jalil F. (2019). How workplace bullying jeopardizes employees’ life satisfaction: the roles of job anxiety and insomnia. *Front. Psychol.* 10:2292. 10.3389/fpsyg.2019.02292 31708827 PMC6821672

[B39] Nielsen M. B. Bjørkelo B. Notelaers G. Einarsen S. (2010a). Sexual harassment: prevalence, outcomes, and gender differences assessed by three different estimation methods. *J. Aggress. Maltreat. Trauma* 19 252–274. 10.1080/10926771003705056

[B40] Nielsen M. B. Matthiesen S. B. Einarsen S. (2010b). The impact of methodological moderators on prevalence rates of workplace bullying. A meta-analysis. *J. Occup. Organ. Psychol.* 83 955–979. 10.1348/096317909x481256 30467716

[B41] Nielsen M. B. Christensen J. O. Finne L. B. Knardahl S. (2020a). Workplace bullying, mental distress, and sickness absence: the protective role of social support. *Intern. Arch. Occup. Environ. Health* 93 43–53. 10.1007/s00420-019-01463-y 31342156

[B42] Nielsen M. B. Notelaers G. Einarsen S. V. (2020b). “Methodological issues in the measurement of workplace bullying,” in *Bullying and Harassment in the Workplace: Theory, Research and Practice*, 3rd Edn, eds Einarsen S. V. Hoel H. Zapf D. Cooper C. L. (Boca Raton, FL: CRC Press), 235–265. 10.1201/9780429462528-8

[B43] Nielsen M. B. Einarsen S. (2012). Outcomes of exposure to workplace bullying: a meta-analytic review. *Work Stress* 26 309–332. 10.1080/02678373.2012.734709

[B44] Nielsen M. B. Einarsen S. V. (2018). What we know, what we do not know, and what we should and could have known about workplace bullying: an overview of the literature and agenda for future research. *Aggress. Viol. Behav.* 42 71–83. 10.1016/j.avb.2018.06.007

[B45] Nielsen M. B. Gjerstad J. Jacobsen D. P. Einarsen S. V. (2017). Does ability to defend moderate the association between exposure to bullying and symptoms of anxiety? *Front. Psychol.* 8:1953. 10.3389/fpsyg.2017.01953 29163321 PMC5682040

[B46] Nielsen M. B. Hetland J. Matthiesen S. B. Einarsen S. (2012). Longitudinal relationships between workplace bullying and psychological distress. *Scand. J. Work Environ. Health* 38 38–46. 10.5271/sjweh.3178 22638759

[B47] Nielsen M. B. Indregard A. R. Krane L. Knardahl S. (2019). Workplace bullying and medically certified sickness absence: direction of associations and the moderating role of leader behavior. *Front. Psychol.* 10:767. 10.3389/fpsyg.2019.00767 31024402 PMC6460766

[B48] Nielsen M. B. Skogstad A. Matthiesen S. B. Glasø L. Aasland M. S. Notelaers G. (2009). Prevalence of workplace bullying in Norway: comparisons across time and estimation methods. *Eur. J. Work Organ. Psychol.* 18 81–101. 10.1080/13594320801969707

[B49] Notelaers G. Einarsen S. (2013). The world turns at 33 and 45: defining simple cutoff scores for the negative acts questionnaire-revised in a representative sample. *Eur. J. Work Organ. Psychol.* 22 670–682. 10.1080/1359432x.2012.690558

[B50] Ólafsson R. F. Jóhannsdóttir H. L. (2004). Coping with bullying in the workplace: the effect of gender, age and type of bullying. *Br. J. Guidan. Counsel.* 32 319–333. 10.1080/03069880410001723549

[B51] Reknes I. Visockaite G. Liefooghe A. Lovakov A. Einarsen S. V. (2019). Locus of control moderates the relationship between exposure to bullying behaviors and psychological strain. *Front. Psychol.* 10:1323. 10.3389/fpsyg.2019.01323 31244725 PMC6563764

[B52] Rosander M. Blomberg S. (2018). *The Whole Picture: Measurement of Psychosocial Work Environment.* Linköping: Linköping University Electronic Press.

[B53] Rosander M. Blomberg S. (2019). Levels of workplace bullying and escalation - a new conceptual model based on cut-off scores, frequency and self-labelled victimization. *Eur. J. Work Organ. Psychol.* 28 769–783. 10.1080/1359432x.2019.1642874

[B54] Salin D. (2001). Prevalence and forms of bullying among business professionals: a comparison of two different strategies for measuring bullying. *Eur. J. Work Organ. Psychol.* 10 425–441. 10.1080/13594320143000771

[B55] Salin D. (2003). The significance of gender in the prevalence, forms and perceptions of bullying. *Nordiske Organisasjonsstudier* 5 30–50.

[B56] Salin D. (2015). Risk factors of workplace bullying for men and women: the role of the psychosocial and physical work environment. *Scand. J. Psychol.* 56 69–77. 10.1111/sjop.12169 25330234

[B57] Salin D. (2018). “Workplace bullying and gender: an overview of empirical findings,” in *Dignity and Inclusion at Work*, eds D’Cruz P. Noronha E. Caponecchia C. Escartín J. Salin D. Tuckey M. R. (Berlin: Springer), 1–31. 10.1007/978-981-10-5338-2_12-1

[B58] Salin D. Lee R. T. Hoel H. (2013). Workplace bullying as a gendered phenomenon. *J. Manag. Psychol.* 28 235–251. 10.1108/02683941311321187

[B59] Salin D. Notelaers G. (2018). The effects of workplace bullying on witnesses: violation of the psychological contract as an explanatory mechanism?. *Intern. J. Hum. Resour. Manag.* 1–21. 10.1080/09585192.2018.1443964 [Epub ahead of print].

[B60] Sidanius J. Pratto F. van Laar C. Levin S. (2004). Social dominance theory: its agenda and method. *Pol. Psychol.* 25 845–880. 10.1111/j.1467-9221.2004.00401.x

[B61] Simpson R. Cohen C. (2004). Dangerous work: the gendered nature of bullying in the context of higher education. *Gender Work Organ.* 11 163–186. 10.1111/j.1468-0432.2004.00227.x

[B62] Spector P. E. Brannick M. T. (2011). Methodological urban legends: the misuse of statistical control variables. *Organ. Res. Methods* 14 287–305. 10.1177/1094428110369842

[B63] Tsuno K. Kawakami N. Tsutsumi A. Shimazu A. Inoue A. Odagiri Y. (2015). Socioeconomic determinants of bullying in the workplace: a national representative sample in Japan. *PLoS One* 10:e0119435. 10.1371/journal.pone.0119435 25751252 PMC4353706

[B64] Vie T. L. Glaso L. Einarsen S. (2011). Health outcomes and self-labeling as a victim of workplace bullying. *J. Psychosom. Res.* 70 37–43. 10.1016/j.jpsychores.2010.06.007 21193099

[B65] Wang M. L. Hsieh Y. H. (2015). Do gender differences matter to workplace bullying? *Work* 53 631–638. 10.3233/WOR-152239 26835866

[B66] Zapf D. Escartìn J. Scheppa-Lahyani M. Einarsen S. V. Hoel H. Vartia M. (2020). “Empirical findings on prevalence and risk groups of bullying in the workplace,” in *Bullying and Harassment in the Workplace: Theory, Research and Practice*, 3rd Edn, eds Einarsen S. V. Hoel H. Zapf D. Cooper C. L. (Boca Raton, FL: CRC Press), 105–162. 10.1201/9780429462528-5

[B67] Zigmond A. S. Snaith R. P. (1983). The hospital anxiety and depression scale. *Acta Psychiatr. Scand.* 67 361–370. 10.1111/j.1600-0447.1983.tb09716.x 6880820

